# A comparison of health-related quality of life between continuous ambulatory peritoneal dialysis and automated peritoneal dialysis in children with stage 5 chronic kidney disease in Thailand: a randomized controlled trial

**DOI:** 10.1007/s00467-024-06632-x

**Published:** 2025-01-20

**Authors:** Montarat Thavorncharoensap, Usa Chaikledkaew, Sitaporn Youngkong, Montira Assanatham, Suwannee Wisanuyotin, Thanaporn Chaiyapak, Natthida Pongwilairat, Konggrapun Srisuwan, Parkpoom Bhummichitra, Patamakom Pruangprasert, Pantipa Boonyapapong, Nawarat Chongchet, Uthaiwan Khongkhanin, Prayong Vachvanichsanong, Wattana Chartapisak, Anirut Pattaragarn

**Affiliations:** 1 Health Technology Assessment Graduate Program, Mahidol University, Bangkok, Thailand; 2 Social and Administrative Pharmacy Excellence Research (SAPER) Unit, Department of Pharmacy, Faculty of Pharmacy, Mahidol University, Bangkok, Thailand; 3 Division of Nephrology, Department of Medicine, Faculty of Medicine Ramathibodi Hospital, Mahidol University, Bangkok, Thailand; 4 Department of Pediatrics, Faculty of Medicine, Khon Kaen University, Khon Kaen, Thailand; 5 Division of Nephrology, Department of Pediatrics, Faculty of Medicine Siriraj Hospital, Mahidol University, Bangkok, Thailand; 6 Department of Pediatrics, Buddhachinaraj Phitsanulok Hospital, Phitsanulok, Thailand; 7 Department of Pediatrics, Phramongkutklao College of Medicine, Bangkok, Thailand; 8 Department of Pediatrics, Surat Thani Hospital, Surat Thani, Thailand; 9 HRH Princess Maha Chakri Sirindhorn Medical Center, Nakhon Nayok, Thailand; 10 Department of Pediatrics, Maharat Nakhon Ratchasima Hospital, Nakhon Ratchasima, Thailand; 11 Department of Pediatrics, Surin Hospital, Surin, Thailand; 12 Department of Pediatrics, Chonburi Hospital, Chonburi, Thailand; 13 Department of Pediatrics, Faculty of Medicine, Prince of Songkla University, Songkla, Thailand; 14 Department of Pediatrics, Faculty of Medicine, Chiang Mai University, Chiang Mai, Thailand

**Keywords:** Automated peritoneal dialysis, Continuous ambulatory peritoneal dialysis, EQ-5D, PedsQL, Quality of life, Chronic kidney disease

## Abstract

**Background:**

Improving health-related quality of life (HRQoL) is one of the main goals in managing stage 5 chronic kidney disease (CKD). However, limited evidence compares HRQoL between continuous ambulatory peritoneal dialysis (CAPD) and automated peritoneal dialysis (APD) in children. This open-label randomized controlled trial (RCT) aimed to compare HRQoL in pediatric patients with stage 5 CKD receiving CAPD vs. APD in Thailand.

**Methods:**

Children with stage 5 CKD were randomized 1:1 to receive APD or CAPD. The primary outcome was HRQoL, measured by EQ-5D-5L, EQ-5D-3L, and PedsQL at baseline, week 16 and week 48. Outcomes were analyzed using linear mixed models.

**Results:**

A total of 60 patients were recruited: 30 with CAPD and 30 with APD. General characteristics, utility scores measured by EQ-5D and HRQoL score measured by PedsQL were comparable between both groups at baseline. During follow-up, no significant differences in terms of utility and HRQoL scores could be identified at week 16 and week 48. Although the children in the APD group seemed to have more favorable changes in some PedsQL domains of PedsQL (school and social domain), as compared to the CAPD group, it was not found that the improvement from baseline was significantly different between both groups.

**Conclusions:**

No significant benefit of APD was found over CAPD in terms of HRQoL improvement. However, larger studies are warranted along with qualitative studies to examine the complete impacts of APD on HRQoL among pediatric patients with stage 5 CKD and their families.

**Graphical abstract:**

**Figure:**
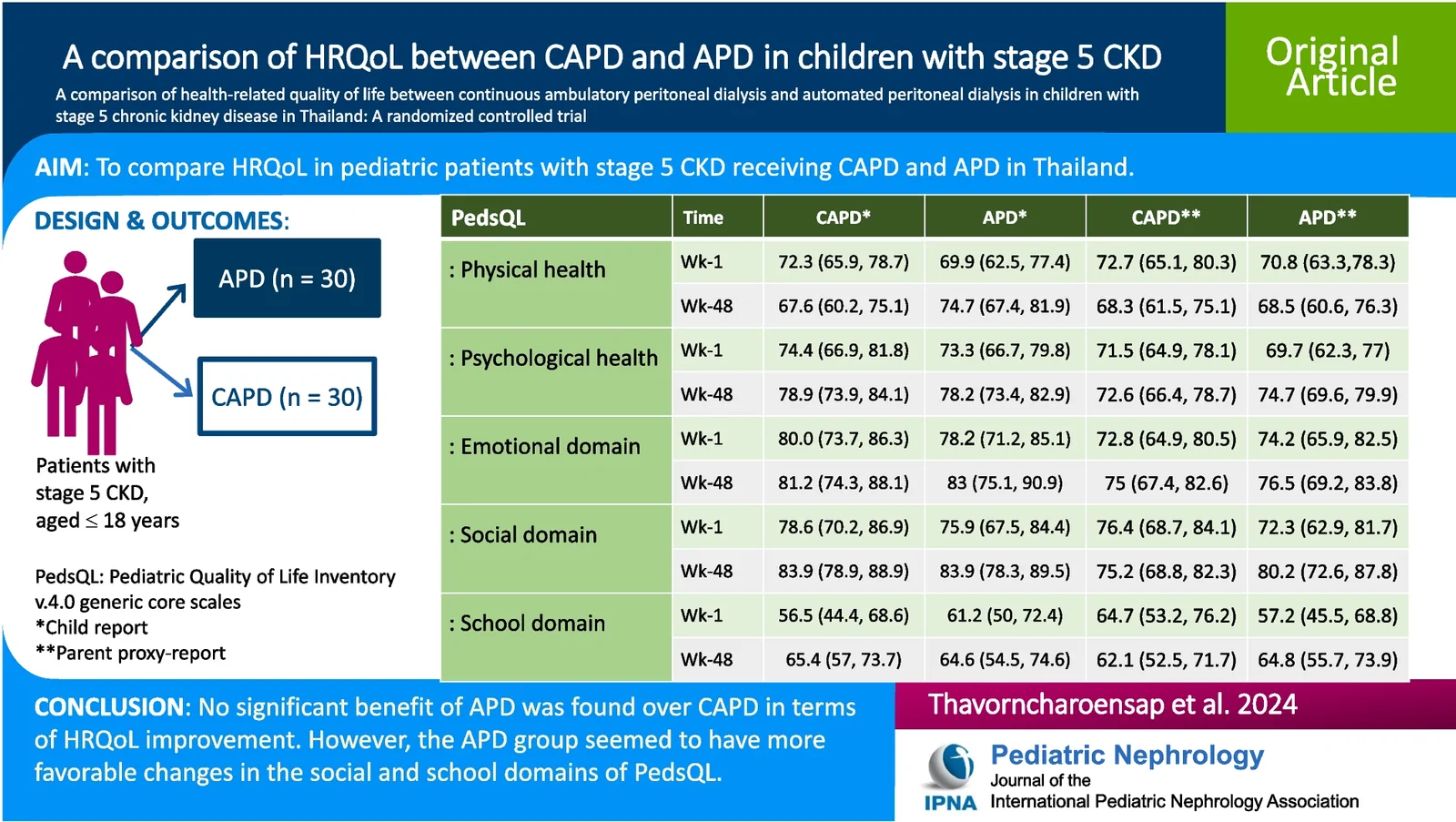
A higher resolution version of the Graphical abstract is available as Supplementary information

**Supplementary Information:**

The online version contains supplementary material available at https://doi.org/10.1007/s00467-024-06632-x.

## Introduction

Stage 5 chronic kidney disease (CKD) and its treatment have a substantial impact on the health-related quality of life (HRQoL) of pediatric patients [1]. Although the debilitating and chronic symptoms of stage 5 CKD lead to limitation of normal functioning and emotional distress, children with stage 5 CKD also experience a massive treatment burden that interferes with school attendance, daily activities, and participation in peer-related activities. Therefore, children with stage 5 CKD have significantly lower HRQoL than their healthy counterparts [2] and even lower than those with other chronic diseases [3].

Peritoneal dialysis (PD) is the most common kidney replacement therapy for children with stage 5 CKD [4]. Currently, there are 2 modalities of PD: continuous ambulatory peritoneal dialysis (CAPD) and automated peritoneal dialysis (APD). Each modality is associated with specific benefits and drawbacks. Compared to CAPD, APD offers greater flexibility by allowing patients to receive their fluid exchange during the night. Additionally, APD can improve patient compliance and is possibly associated with a lower incidence of peritonitis and lower intraperitoneal pressure [5]. In contrast, APD is associated with higher costs, possibly inadequate sodium removal, and disturbed sleep [5]. In terms of PD survival, a recent systematic review [6] reported no significant difference between APD and CAPD.

Although HRQoL is recognized as an important indicator to determine the success of the treatment/ intervention and is found to be a predictive indicator of mortality and hospitalization among patients with stage 5 CKD [7, 8], very few studies are available that examine HRQoL among children with stage 5 CKD undergoing PD [2, 3, 9]. Although APD appears to improve HRQoL, as it allows patients to spend their day on normal activities while performing PD during the night, current evidence comparing HRQoL between adult patients undergoing APD and CAPD is inconclusive. Although the previous meta-analysis found that adult patients treated with APD have significantly higher HRQoL than those treated with CAPD [10], several updated studies did not find significant differences between the 2 groups [11–13]. Furthermore, one study even found a significantly lower HRQoL among APD patients [14]. In particular, almost all evidence was based on observational studies and not randomized controlled trials (RCT). Evidence comparing CAPD and APD in terms of HRQoL is even less available among pediatric patients [15].

In Thailand, the National Health Security Office (NHSO) has implemented the 'PD-First policy', which allows full reimbursement for PD among patients under the Universal Health Coverage Scheme (UHC) since 2008. Later in February 2023, NHSO allowed beneficiaries to freely choose between PD and hemodialysis (HD). However, only CAPD but not APD is included in the reimbursement list for the UHC beneficiary. Unlike many western countries, the use of APD in Thailand is quite limited. This RCT is part of a research project aiming to determine feasibility in establishing the APD service in the UHC system for Thai children with stage 5 CKD. The main objective of this RCT was to compare HRQoL of children with stage 5 CKD who underwent APD with those who underwent CAPD. The results of the study will serve as essential evidence to determine the success of each modality of PD treatment and to provide HRQoL information, which is required to perform cost-utility analysis to inform policy decisions on APD coverage in the country.

## Methods

### Trial design

The study was an open-labeled, multicenter two-group clinical trial. The trial was registered on ClinicalTrials.gov under the following identifier: TCTR20200810001. The trial was conducted in accordance with the Good Clinical Practice guidelines and the principles of the Declaration of Helsinki. Ethics approval was obtained from the Central Research Ethics Committee (CREC): COA-CREC074/2020. Written informed consent was obtained from all parents or legally acceptable representatives and verbal informed assent was obtained from all participants before any research procedures started.

### Participants

The patients were recruited from 11 public tertiary care hospitals in Thailand. During the recruitment period from 8 September 2020 to 26 December 2021, medical records of children with stage 5 CKD receiving treatment in the nephrology clinic of the participating hospitals were screened for eligibility. Patients were eligible for inclusion in the study if they met the following criteria: 1) aged 1 to 18 years old; 2) diagnosed with stage 5 CKD (an estimated glomerular filtration rate (GFR) of less than 15 ml/minute 1.73 m^2^ of body surface area). The estimated GFR was determined using the Schwartz equation [16]; and 3) had undergone PD for at least 1 month. The exclusion criteria were as follows: 1) lack of access to electricity; 2) could not communicate in Thai; and 3) scheduled for kidney transplant within the next 6 months. Withdrawal criteria included: 1) patients switched from PD to HD for more than 1 month; 2) patients switched from CAPD to APD or vice versa for more than 1 month; 3) patients stopped PD before undergoing kidney transplant; 4) patients requested to withdraw; and 5) death.

Patients who met the eligibility criteria were invited to participate in the study. The patients who consented were stratified by co-morbidity status (i.e., co-morbidity, no co-morbidity) and randomly assigned in a 1:1 ratio to receive CAPD or APD using online software provided by Castor EDC [17]. A permuted block randomization with a block size of 4 was performed. Web-based randomization was performed for concealment of allocation.

### Interventions

#### APD group

During the first week, the study nurses trained patients and caregivers on the use of APD. APD was performed as continuous cycle peritoneal dialysis (CCPD) with a dialysis solution, which contained 1.5% dextrose W/V. There were 5 to 10 exchanges at night with 600–800 or 1,000–1,200 ml/m^2^ of body surface area/exchange for patients aged less than 2 or ≥ 2 years, respectively. The length of each exchange was between 1 and 3 h. During the day, the peritoneal cavity was filled with a dialysis solution volume of 50–100 percentages of each night cycle.

#### CAPD group

Patients who were assigned to CAPD continued their usual CAPD. During the first week, the study nurse provided a training session for patients with CAPD and caregivers and evaluated the technique. Dialysis solution contained 1.5% dextrose W/V. There were 3 to 5 exchanges per day and 1 exchange during the night. The exchange volumes were 600–800 ml/m^2^ or 1,000–1,200 ml/m^2^ of body surface area per exchange for patients aged less than 2 years or ≥ 2 years, respectively, identical to the night-time exchange volumes in the APD group. The length of each cycle was between 4–6 h.

For both groups, adjustment of the dialysis volume, number of exchanges and the length of each exchange was determined based on the state of body volume, urine output, dietary and water intake, and uremic toxin. In the event that patients experienced inflow/outflow pain in both groups, tidal peritoneal dialysis (TPD) could be performed during the first week.

During the first week of enrollment, study nurses, together with dieticians and pharmacists, provided counseling on PD self-management to both groups. Booklets which covered important information (i.e., fluid balance, dietary care, medication use, dental hygiene, common problems and solutions) were also provided to both groups. During the first month of enrollment, study nurses followed up with patients/ caregivers weekly to ensure that they could perform PD properly.

### Outcomes

The primary endpoint was utility scores, measured by EQ-5D-5L using parents as a proxy. Secondary endpoints included utility scores, measured by EQ-5D-3L using parents as a proxy, utility scores measured by EQ-5D-5L from children, and HRQoL scores measured by PedsQL™ 4.0 Generic Core Scales (both parent proxy and child report). Details on the instruments used in the data collection are described in Table 1.

**Table 1:** Instruments used to assess HRQoL

Age of the patient	Instruments	Interviewee	
		Parent	Child
2–4 years	EQ-5D-5L	X	
	EQ-5D-3L	X	
	PedsQL Parent proxy-reported	X	
5–7 years	EQ-5D-5L	X	
	EQ-5D-3L	X	
	PedsQL Child-self-reported		X
	PedsQL Parent proxy-reported	X	
8–12 years	EQ-5D-5L	X	
	EQ-5D-3L	X	
	EQ-5D-5L		X
	PedsQL Child-self-reported		X
	PedsQL Parent proxy-reported	X	
> 12 years	EQ-5D-5L	X	
	EQ-5D-3L	X	
	EQ-5D-5L		X
	PedsQL Child-self-reported		X
	PedsQL Parent proxy-reported	X	

EQ-5D (3L and 5 L) is among the most frequently used generic preference-based measures [18]. It consists of a short descriptive system questionnaire and a visual analog scale (EQ-VAS). The descriptive system of EQ-5D comprises 5 dimensions, namely mobility, self-care, usual activities, pain/discomfort and anxiety/depression [19]. For EQ-5D-5L, each dimension has five response levels of severity (i.e., no problems, slight problems, moderate problems, severe problems, unable to/extreme problems). On the other hand, each dimension of EQ-5D-3L has only 3 response levels of severity (i.e., no problems, some problems, extreme problems). An EQ-5D summary index or utility can be derived by applying a formula that attaches values (weights) to each of the levels in each dimension. Utilities can be used as quality adjustment weights for quality adjusted life years (QALY), which is often used in economic evaluation. Utility could range from −1 to 1, corresponding to the worst health state and the best health state, respectively. As 0 represents death, the negative values for utility represent the health states that were worse than death. For EQ-VAS, respondents were asked to indicate their overall health today on a 0—100 vertical scale, where 0 means the worst imaginable health state and 100 means the best imaginable health state. EQ-5D-3L and EQ-5D-5L could be used among children ≥ 12 years of age [19]. The utility values were derived based on the EQ-5D-3L and EQ-5D-5L Thai value set [20, 21]. A higher utility score represents a better HRQoL. The ranges of the utility score derived from EQ-5D-5L and EQ-5D-3 were −0.4212 to 1 [20] and −0.454 to 1 [21], respectively.

PedsQL™ 4.0 Generic Core Scales is a generic instrument [22, 23], evaluating HRQoL of children using a youth self-report and parent proxy report. This 23-item questionnaire comprises 4 dimensions: physical functioning (8 items), emotional functioning (5 items), social functioning (5 items) and functioning at school (5 items) with a Likert-type response scale ranging from 0 = never to 4 = almost always). The scale has 4 versions according to the age of the child: 2 − 4 years, 5–7 years, 8–12 years, and 13–18 years [23]. The PedsQL™ 4.0 Generic Core Scales are scored on a scale of 0 to 100, with higher numbers correlating with a better quality of life [23]. The scores were reported for each dimension along with the physical health summary score, a psychosocial health summary score, and a total score. The instrument was translated and validated in Thailand [22].

Other secondary outcomes include peritonitis and other infections related to PD, residual kidney function, peritoneal membrane transport function, complications related to PD and stage 5 CKD (i.e. electrolyte disorders; hypo/hypertension; hernia; fluid leak), and sleep disturbance. It should be noted that the analysis for secondary outcomes other than HRQoL will be reported separately in other publications.

### Sample size

Based on the standard deviation of 0.23 from a previous study [14], we needed to enroll 37 patients per group to detect a between-group difference in the utility score measured by EQ-5D-5L as small as 0.15 points (power of 80%, two-sided significant level of 5%, 1:1 allocation ratio). This difference was derived from the previous literature that found that the mean minimal clinical important difference (MCID) of utility derived from EQ-5D was 0.18 [24] and that the MCID for the EQ-5D estimates for dialysis patients ranged from 0.034 to 0.158, depending on the method [25]. Furthermore, the previous study found that the difference in utility values between the 2 groups was around 0.16 [11].

### Data collection

The patients were required to follow up at week 4, 8, 16, 24, 32, 40, and 48. Primary outcomes were assessed by face-to-face interviews with trained nurses at the beginning of the study (1 week after randomization and training for PD was provided), and at week 16 and week 48 of follow-up.

### Statistical analysis

All analyses were performed using Stata version 17.0 (StataCorp). Descriptive statistics were used to characterize the sample, including means and standard deviations, or medians and interquartile ranges for continuous variables, with frequencies and percentages calculated for categorical variables. HRQoL scores at baseline (week 1) were compared between the CAPD and APD groups using Wilcoxon rank sum tests as the assumption of normality was not satisfied. A linear mixed model was used to assess the change in HRQoL scores between children randomly assigned to APD vs. CAPD. The covariance structure was defined as unstructured. The main effect of time and the interaction effect between time and intervention group (time*intervention group) were added to the model, as shown in Eq. 1.

1 $$\mathrm{Y}={\beta }_{0}+{\beta }_{1} \mathrm{time}+{\beta }_{2} \mathrm{time}*\text{intervention group}+{\varepsilon }_{i}$$

Where Y represents the HRQoL score, βs represent regression coefficients, and ε represents an error term. When the intervention group = 0 (APD), β_1_ represents the change in HRQoL score by time in the APD group. On the other hand, when the intervention group = 1 (CAPD), β_1_ + β_2_ represents the change in HRQoL score by time in the CAPD group. Therefore, β_2_ represents the difference in the change in HRQoL score over time between the CAPD and APD groups. Both the per protocol (PP) analysis and modified intention-to-treat (ITT) analysis were conducted. For modified ITT, patients with information on the outcome at baseline were included. Missing data were imputed using the last observation carried forward method.

## Results

**Fig. 1:**
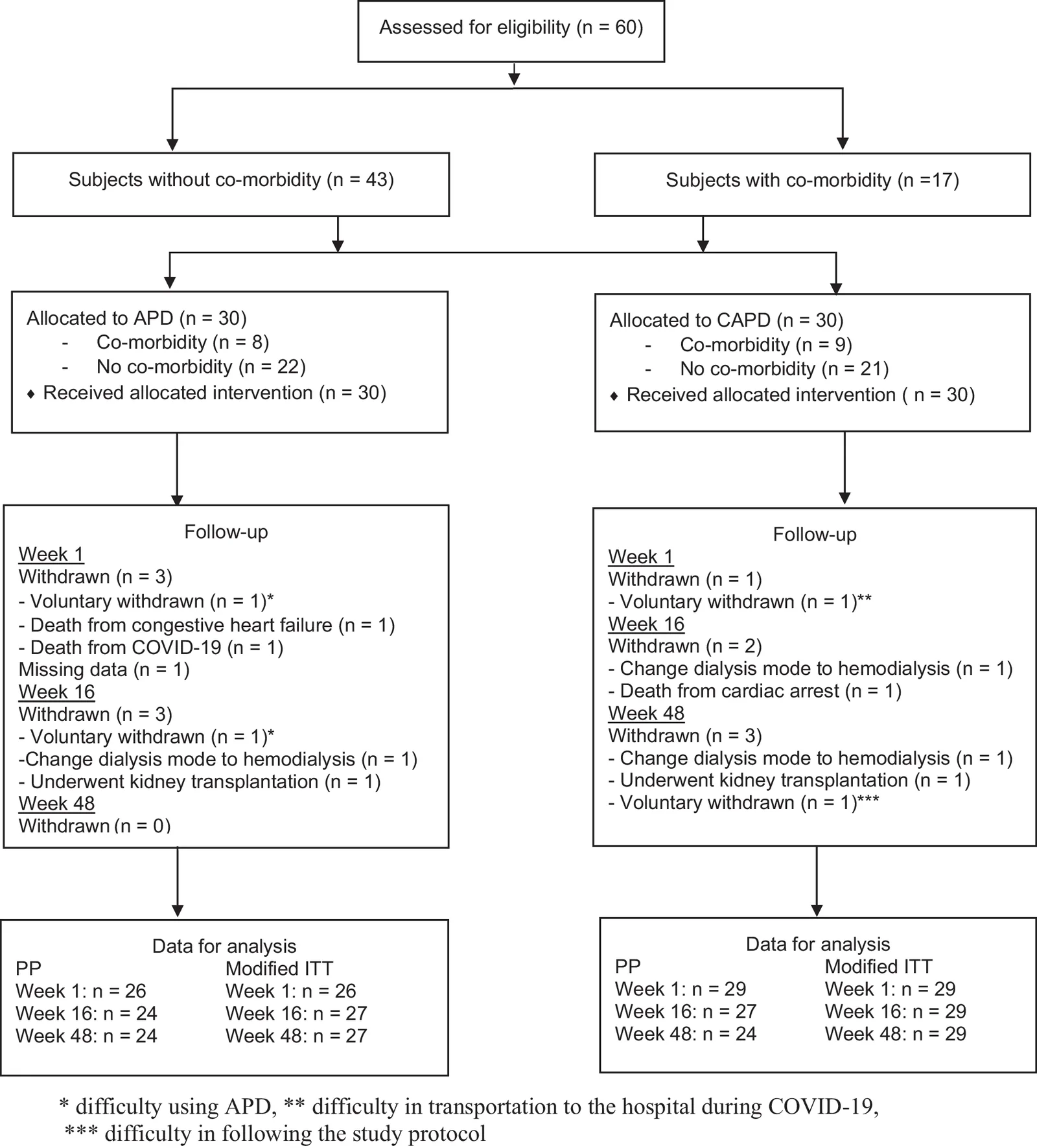
Flow of participant recruitment, randomization and follow-up

The flow of participants is reported in Fig. 1. Of the 60 eligible patients, 30 each were randomly assigned to APD and CAPD. For the APD group, 6 were withdrawn from the study (i.e., death from COVID (*n* = 1), death from congestive heart failure (*n* = 1), voluntary withdrawal due to difficulty using APD (*n* = 2), move to HD (*n* = 1), and underwent kidney transplantation (*n* = 1). For the CAPD group, 6 were excluded from the study (i.e., underwent kidney transplantation (*n* = 1), death from cardiac arrest (*n* = 1), moved to HD (*n* = 2), and voluntary withdrawal (*n* = 2). Of the patients in the CAPD group that voluntarily withdrew, one was due to difficulty in participating during the COVID-19 pandemic. The other voluntary withdrawal was due to the difficulty in following the protocol. The baseline characteristics of the participants are shown in Table 2. At baseline, there was no imbalance in important demographic and clinical characteristics (i.e., age, sex, level of education, hospital settings, duration of CKD and duration of PD) between the two groups.

**Table 2:** Baseline characteristics of the patients

	Median (IQR) or N (%)		*P* value^a^
	CAPD (*n* = 30)	APD (*n* = 30)	
Age (year)	13.0 (4.3)	11.0 (6.5)	0.10
Age (year)			
2–4	1 (3.3%)	4 (13.3%)	0.35
5–7	1 (3.3%)	3 (10.0%)	
8–12	12 (40.0%)	10 (33.3%)	
> 12	16 (53.3%)	13 (43.3%)	
Gender			0.79
Male	16 (53.3%)	15 (50%)	
Female	14 (46.7%)	15 (50%)	
Education			0.44
Too young to attend school	1 (3.8%)	5 (16.7%)	
Kindergarten	1 (3.8%)	3 (10.0%)	
Primary school	16 (61.5%)	15 (50%)	
Secondary school (Grade 7–9)	6 (23.1%)	6 (20%)	
Secondary school (Grade 10–12)	2 (7.7%)	1 (3.3%)	
Hospital settings			0.78
University hospital	21 (70%)	20 (66.7%)	
Non-university tertiary hospital	9 (30%)	10 (33.3%)	
Time since diagnosis of CKD (year)	1.69 (2.67)	1.28 (2.2)	0.79
Time on dialysis (year)	1.25 (1.94)	1.24 (2.03)	0.69
Comorbidities	9 (30%)	8(26.7%)	0.77

^a^Wilcoxon Signed Ranks Test / Chi-square Test

Table 3 presents HRQoL scores assessed by parents of the 2 groups at week 1, week 16, and week 48. When comparing the APD and CAPD groups, no significant differences in all scores were identified at week 1. The HRQoL scores assessed by the children of the 2 groups are presented in Table 4. Similarly to those evaluated by parents, no significant differences were identified between the 2 groups in terms of HRQoL scores at week 1.

**Table 3:** HRQoL scores assessed by parent

HRQoL assessment	PP analysis				Modified ITT analysis			
	No. of patients		Mean (95% CI)		No. of patients		Mean (95% CI)	
	CAPD	APD	CAPD	APD	CAPD	APD	CAPD	APD
PedsQL: Physical health summary score								
week-1	29	26	72.70 (65.07, 80.33)	70.81 (63.31, 78.31)	29	26	72.70 (65.07, 80.33)	70.81 (63.31,78.31)
week-16	27	24	77.45 (70.89, 84.00)	75.99 (66.26, 85.71)	29	27	76.09 (69.67, 82.52)	76.11 (67.51, 84.71)
week-48	24	24	68.59 (60.65, 76.52)	67.40 (58.61, 76.18)	29	27	68.29 (61.49, 75.09)	68.47 (60.61, 76.34)
PedsQL: Psychological health summary score								
week-1	29	26	71.55 (64.99, 78.12)	69.68 (62.32, 77.03)	29	26	71.55 (64.99, 78.12)	69.67 (62.32, 77.03)
week-16	27	24	77.28 (70.40, 84.16)	79.61 (72.73, 86.48)	29	27	75.69 (68.90, 82.48)	78.29 (72.04, 84.54)
week-48	24	24	75.21 (68.95, 81.47)	75.63 (69.86, 81.39)	29	27	72.59 (66.41, 78.75)	74.75 (69.56, 79.94)
PedsQL: Emotional domain score								
week-1	29	26	72.76 (64.99, 80.53)	74.23 (65.98, 82.48)	29	26	72.76 (64.99, 80.53)	74.23 (65.97, 82.48)
week-16	27	24	77.41 (69.62, 85.19)	80.63 (72.82, 88.43)	29	27	75.86 (68.30. 83.41)	79.26 (71.99, 86.53)
week-48	24	24	77.29 (69.59, 84.99)	77.50 (69.60, 85.40)	29	27	75.00 (67.36, 82.64)	76.48 (69.21, 83.76)
PedsQL: Social domain score								
week-1	28	26	76.43 (68.75, 84.11)	72.31 (62.95, 81.66)	29	26	76.43 (68.75, 84.11)	72.31 (62.95, 81.66)
week-16	25	24	81.00 (73.53, 88.47)	82.08 (74.56, 89.60)	29	27	80.68 (73.61, 87.76)	82.04 (75.09, 88.98)
week-48	24	24	77.92 (70.69, 85.15)	80.00 (71.67, 88.33)	29	27	75.21 (68.76, 82.27)	80.19 (72.56, 87.82)
PedsQL: School domain score								
week-1	20	16	64.75 (53.25, 76.25)	57.19 (45.55, 68.82)	20	16	64.75 (53.25, 76.25)	57.19 (45.55, 68.82)
week-16	17	15	71.76 (59.27, 84.26)	73.00 (60.32, 85.68)	20	19	67.5 (55.75, 79.25)	65.00 (52.14, 77.85)
week-48	15	20	65.67 (54.07, 77.27)	69.50 (61.58, 77.42)	21	23	62.14 (52.55, 71.73)	64.78 (55.69, 73.87)
PedsQL: Total score								
week-1	29	26	71.89 (65.42, 78.36)	70.04 (63.56, 76.52)	29	26	71.89 (65.42, 78.36)	70.04 (63.56, 76.51)
week-16	27	24	77.36 (71.37, 83.30)	78.08 (70.64, 85.52)	29	27	75.84 (69.89, 81.80)	77.35 (70.27, 83.97)
week-48	24	24	72.45 (66.49, 78.40)	72.63 (66.30, 78.96)	29	27	70.78 (65.23, 76.34)	72.50 (66.90, 78.09)
EQ-5D-5L								
week-1	29	26	0.86 (0.82, 0.90)	0.83 (0.74, 0.91)	29	26	0.86 (0.82, 0.90)	0.83 (0.75, 0.91)
week-16	27	23	0.89 (0.85, 0.94)	0.82 (0.71, 0.93)	29	27	0.89 (0.84, 0.93)	0.83 (0.74, 0.93)
week-48	24	24	0.87 (0.83, 0.91)	0.84 (0.74, 0.95)	29	27	0.87 (0.83, 0.90)	0.85 (0.76, 0.95)
EQ-5D-5L VAS								
week-1	29	26	0.84 (0.78, 0.90)	0.83 (0.76, 0.89)	29	26	0.84 (0.78, 0.90)	0.83 (0.77, 0.89)
week-16	27	23	0.86 (0.82, 0.90)	0.84 (0.78, 0.91)	29	27	0.85 (0.80, 0.89)	0.86 (0.80, 0.91)
week-48	24	24	0.83 (0.77, 0.89)	0.88 (0.82, 0.93)	29	27	0.83 (0.78, 0.89)	0.88 (0.83, 0.93)
EQ-5D-3L								
week-1	29	26	0.67 (0.60, 0.75)	0.70 (0.59, 0.81)	29	26	0.67 (0.60, 0.75)	0.70 (0.59, 0.81)
week-16	27	24	0.75 (0.65, 0.85)	0.70 (0.56, 0.84)	29	27	0.73 (0.63, 0.83)	0.71 (0.58, 0.83)
week-48	23	24	0.68 (0.56, 0.80)	0.75 (0.61, 0.89)	29	27	0.67 (0.57, 0.78)	0.75 (0.62, 0.87)
EQ-5D-3L VAS								
week-1	29	26	0.83 (0.77, 0.89)	0.83 (0.77, 0.89)	29	26	0.83 (0.77, 0.89)	0.83 (0.77, 0.89)
week-16	26	24	0.87 (0.82, 0.91)	0.87 (0.82, 0.92)	29	27	0.85 (0.50, 0.90)	0.87 (0.83, 0.92)
week-48	23	24	0.84 (0.77, 0.91)	0.86 (0.80, 0.91)	29	27	0.84 (0.77, 0.90)	0.87 (0.81, 0.92)

**Table 4:** HRQoL scores assessed by children

HRQoL assessment	PP analysis				Modified ITT analysis			
	No. of patients		Mean (95% CI)		No. of patients		Mean (95% CI)	
	CAPD	APD	CAPD	APD	CAPD	APD	CAPD	APD
PedsQL: Physical health summary score								
week-1	28	22	72.32 (65.95, 78.70)	69.94 (62.49, 77.39)	28	22	72.32 (65.95, 78.70)	69.93 (62.49, 77.39)
week-16	27	20	75.24 (69.62, 80.87)	79.31 (72.60, 86.02)	28	23	74.78 (69.30, 80.28)	77.12 (70.18, 84.05)
week-48	24	20	66.66 (58.27, 75.04)	76.51 (69.17, 83.85)	28	23	67.62 (60.17, 75.07)	74.68 (67.42, 81.94)
PedsQL: Psychological health summary score								
week-1	28	22	74.38 (66.98, 81.77)	73.30 (66.76, 79.83)	28	22	74.38 (66.98, 81.77)	73.30 (66.75, 79.83)
week-16	27	20	77.13 (71.20, 83.06)	82.79 (77.52, 88.06)	28	23	76.51 (70.68, 82.36)	81.84 (76.80, 88.90)
week-48	24	20	78.56 (72.87, 84.24)	78.63 (73.59, 83.66)	28	23	78.97 (73.95, 84.15)	78.22 (73.45, 82.99)
PedsQL: Emotional domain score								
week-1	28	22	80.00 (73.67, 86.33)	78.18 (71.22, 85.14)	28	22	80.00 (73.67, 86.33)	78.18 (71.22, 85.14)
week-16	27	20	82.78 (76.59, 88.97)	83.50 (75.90, 91.10)	28	23	82.68 (76.72, 88.64)	81.52 (74.43, 88.62)
week-48	24	20	80.21 (72.30, 88.12)	85.25 (76.71, 93.79)	28	23	81.25 (74.35, 88.14)	83.04 (75.12, 90.97)
PedsQL: Social domain score								
week-1	28	22	78.57 (70.25, 86.90)	75.91 (67.46, 84.36)	28	22	78.57 (70.25, 86.90)	75.90 (67.46, 84.36)
week-16	27	20	79.81 (73.50, 86.13)	90.75 (86.18, 95.32)	28	23	79.29 (73.11, 85.46)	88.91 (83.70, 94.13)
week-48	24	20	83.54 (78.17, 88.91)	85.00 (79.47, 90.53)	28	23	83.93 (78.99, 88.86)	83.91 (78.31, 89.52)
PedsQL: School domain score								
week-1	20	17	56.50 (44.37, 68.63)	61.18 (50.03, 72.33)	20	17	56.50 (44.37, 68.63)	61.18 (50.03, 72.38)
week-16	17	16	66.18 (54.91, 77.44)	69.38 (62.30, 76.45)	20	18	65.75 (55.56, 75.94)	67.61 (58.58, 76.65)
week-48	15	18	65.22 (55.15, 75.29)	64.74 (55.32, 74.16)	20	18	65.42 (57.01, 73.73)	64.57 (54.53, 74.59)
PedsQL: Total score								
week-1	28	22	73.27 (67.20, 79.35)	71.84 (66.14, 77.54)	28	22	73.27 (67.20, 79.35)	71.84 (66.14, 77.53)
week-16	27	20	76.05 (71.37, 80.73)	81.48 (76.29, 86.67)	28	23	75.51 (70.87, 80.14)	80.12 (75.06, 85.17)
week-48	24	20	73.95 (67.77, 80.13)	77.91 (72.81, 83.01)	28	23	74.53 (68.99, 80.06)	77.00 (72.16, 81.85)
EQ-5D-5L								
week-1	27	20	0.87 (0.81, 0.94)	0.89 (0.81, 0.96)	27	20	0.87 (0.81, 0.94)	0.89 (0.82, 0.96)
week-16	26	17	0.92 (0.88, 0.95)	0.95 (0.90, 1.01)	27	20	0.91 (0.87, 0.95)	0.96 (0.91, 1.00)
week-48	23	17	0.89 (0.85, 0.93)	0.94 (0.89, 0.98)	27	20	0.89 (0.86, 0.93)	0.94 (0.90, 0.98)
EQ-5D-5 l VAS								
week-1	27	20	0.85 (0.78, 0.92)	0.83 (0.75, 0.91)	27	20	0.85 (0.78, 0.92)	0.83 (0.75, 0.91)
week-16	26	17	0.86 (0.80, 0.92)	0.83 (0.74, 0.91)	27	20	0.86 (0.81, 0.92)	0.85 (0.77, 0.92)
week-48	23	17	0.89 (0.83, 0.95)	0.86 (0.79, 0.93)	27	20	0.90 (0.85, 0.95)	0.87 (0.81, 0.94)

When looking at the change from week 1, it should be noted that the mean (95% CI) for the school domain scores assessed by parents increased from 57.19 (45.55, 68.82) at week 1 to 64.78 (55.69, 73.87) at week 48 in the APD group while it decreased from 64.75 (53.25, 76.25) to 62.14 (52.55, 71.73) in the CAPD group. Furthermore, the mean (95% CI) for the social domain score assessed by the parent increased from 72.31 (62.95, 81.66) at week 1 to 80.19 (72.56, 87.82) at week 48 in the APD group, compared to the reduction of 76.43 (68.75, 84.11) to 75.21 (68.76, 82.27) in the CAPD group.

For the scores that were directly measured in children, the mean utility score (95% CI) measured by EQ-5D-5L increased from 0.89 (0.82, 0.96) at week 1 to 0.94 (0.90, 0.98) at week 48 in the APD group, while it increased from 0.87 (0.81, 0.94) to 0.89 (0.86, 0.93) in the CAPD group. For PedsQL, we found that the mean score (95% CI) for the physical domains evaluated by the children increased from 69.93 (62.49, 77.39) at week 1 to 74.68 (67.42, 81.94) at week 48 in the APD group, while it decreased from 72.32 (65.95, 78.70) to 67.62 (60.17, 75.07) in the CAPD group. Furthermore, the mean score (95% CI) for the emotional domains assessed by the children increased from 78.18 (71.22, 85.14) at week 1 to 83.04 (75.12, 90.97) at week 48 in the APD group, while it appeared stable for the CAPD group. Similarly, the score for the social domain assessed by children increased from 75.90 (67.46, 84.36) at week 1 to 83.91 (78.31, 89.52) at week 48 in the APD group, while it increased from 78.57 (70.25, 86.90) to 83.93 (78.99, 88.86) in the CAPD group.

However, no significant differences were identified in terms of the change in utility scores and HRQoL scores from week 1 between the two groups, as indicated by the non-significant coefficients of the interaction term between time and the intervention group (Tables 5 and 6).

**Table 5:** Comparison of HRQoL change at 48 weeks between patients receiving CAPD and APD (ITT analysis)

QOL assessment	Time (modified ITT analysis)		Interaction group*time (modified ITT analysis)	
	Coefficient (95%CI)	*P* value^a^	Coefficient (95%CI)	*P* value^a^
Parent-Proxy				
EQ-5D-5L	0.0005 (−0.001, 0.002)	0.400	−0.0004 (−0.002, 0.001)	0.482
EQ-5D-5L VAS	0.0008 (−0.001, 0.002)	0.217	−0.001 (−0.002, 0.001)	0.170
EQ-5D-3L	0.0012 (−0.0012, 0.0036)	0.315	−0.001 (−0.004, 0.001)	0.310
EQ-5D-3L VAS	0.0004 (−0.001, 0.002)	0.609	−0.0005 (−0.002, 0.001)	0.511
PedsQL: Physical health summary score	−0.12 (−0.31, 0.06)	0.182	−0.01 (−0.21, 0.19)	0.914
PedsQL: Psychological health summary score	0.06 (−0.06, 0.18)	0.306	−0.05 (−0.19, 0.09)	0.509
PedsQL: Emotional domain score	0.02 (−0.15, 0.19)	0.806	−0.004 (−0.20, 0.19)	0.970
PedsQL: Social domain score	0.09 (−0.07, 0.25)	0.281	−0.12 (−0.31, 0.06)	0.203
PedsQL: School domain score	0.09 (−0.09, 0.27)	0.356	−0.10 (−0.29, 0.10)	0.348
PedsQL: Total score	−0.01 (−0.14, 0.11)	0.847	−0.04 (−0.18, 0.10)	0.583
Children				
EQ-5D-5L	0.0005 (−0.001, 0.002)	0.383	−0.001(−0.002, 0.001)	0.063
EQ-5D-5L VAS	0.0005 (−0.001, 0.002)	0.484	0.0005 (−0.001, 0.002)	0.530
PedsQL: Physical health summary score	0.0653 (−0.1008, 0.2314)	0.441	−0.16 (−0.35, 0.03)	0.095
PedsQL: Psychological health summary score	−0.0007 (−0.107, 0.106)	0.990	0.07 (−0.04, 0.19)	0.206
PedsQL: Emotional domain score	0.12 (0.01, 0.23)	0.03	−0.08 (−0.22, 0.05)	0.243
PedsQL: Social domain score	0.04 (−0.10, 0.17)	0.602	0.08 (−0.06, 0.22)	0.262
PedsQL: School domain score	−0.004 (−0.205, 0.196)	0.966	0.01 (−0.22, 0.24)	0.931
PedsQL: Total score	0.029 (−0.084, 0.141)	0.618	−0.02 (−0.15, 0.10)	0.719

^a^Linear mixed model, Covariance pattern: Unstructured

**Table 6:** Comparison of HRQoL change at 48 weeks between patients receiving CAPD and APD (PP analysis)

QOL assessment	Time (PP analysis)		Interaction group*time (PP analysis)	
	Coefficient (95%CI)	*P* value^a^	Coefficient (95%CI)	*P* value^a^
Parent-Proxy				
EQ-5D-5L	0.0005 (−0.0005, 0.001)	0.313	−0.0004 (−0.002, 0.001)	0.574
EQ-5D-5L VAS	0.0008 (−0.0003, 0.002)	0.156	−0.0009 (−0.002, 0.001)	0.261
EQ-5D-3L	0.001 (−0.001, 0.003)	0.264	−0.001 (−0.005, 0.002)	0.396
EQ-5D-3L VAS	0.0004 (−0.001, 0.002)	0.476	−0.0003 (−0.002, 0.001)	0.754
PedsQL: Physical health summary score	−0.11 (−0.27, 0.06)	0.203	0.01 (−0.21, 0.24)	0.905
PedsQL: Psychological health summary score	0.03 (−0.08, 0.14)	0.561	−0.04 (−0.19, 0.11)	0.630
PedsQL: Emotional domain score	0.001 (−0.15, 0.15)	0.988	0.01 (−0.21, 0.22)	0.960
PedsQL: Social domain score	0.07 (−0.08, 0.212)	0.386	−0.11 (−0.31, 0.09)	0.289
PedsQL: School domain score	0.04 (−0.12, 0.21)	0.619	−0.11 (−0.33, 0.11)	0.338
PedsQL: Total score	−0.02 (−0.14, 0.09)	0.696	−0.03 (−0.19, 0.13)	0.746
Children				
EQ-5D-5L	0.0005 (−0.001, 0.001)	0.342	−0.0009 (−0.002, 0.0001)	0.091
EQ-5D-5L VAS	0.0007 (−0.001, 0.002)	0.301	0.001 (−0.001, 0.002)	0.470
PedsQL: Physical health summary score	0.04 (−0.11, 0.18)	0.639	−0.21 (−0.42, 0.01)	0.056
PedsQL: Psychological health summary score	−0.01 (−0.10, 0.09)	0.852	0.07 (−0.06, 0.19)	0.298
PedsQL: Emotional domain score	0.09 (−0.01, 0.19)	0.070	−0.12 (−0.27, 0.03)	0.117
PedsQL: Social domain score	0.01 (−0.12, 0.13)	0.902	0.05 (−0.10, 0.19)	0.548
PedsQL: School domain score	0.03 (−0.122, 0.21)	0.768	0.05 (−0.20, 0.30)	0.712
PedsQL: Total score	0.01 (−0.01, 0.11)	0.869	−0.05 (−0.19, 0.10)	0.518

^a^Linear mixed model, Covariance pattern: Unstructured

## Discussion

To our knowledge, this is the first RCT to compare the effect of CAPD and APD on HRQoL in children with stage 5 CKD. Although children in the APD group appeared to have a more favorable change in some domains, such as school and social domains, compared to the CAPD group, no significant differences could be identified. However, it should be noted that the magnitude of the difference between the 2 groups seemed to be larger than the MCID of the PedsQL questionnaire in particular domains [26].

We found that the mean change at week 48 from baseline in the school scores domain measured by PedsQL (parent proxy) in the APD group was greater than that of the CAPD group (7.59 vs. −2.61). In addition, the magnitude of the change in the APD group was greater than the MCID of 5.71 [26]. For the PedsQL scores measured by children, we found that the mean change at week 48 from baseline in the social domain of the APD group was greater than that of the CAPD group (8.01 vs. 5.36), and was equal to the MCID of 8.01 [26]. Regarding the total score of PedsQL measured by children, we also observed that the mean change in the APD group at baseline was greater than that of the CAPD group (5.16 vs. 1.26) and the MCID of 4.4 [27]. When looking at the mean change in utility values, the most recent study indicated that the MCID of EQ-5D-5L for improvement among dialysis patients ranged between 0.037 and 0.122 [28]. Generally, we could not identify the significant trend of improvement in the utility scores of EQ-5D-5L in both groups of patients.

The main reason for the non-statistically significant difference while the magnitude of difference seemed to be larger than MCID for some domains could be possibly due to the small sample size. Due to the limited number of children with stage 5 CKD and the COVID-19 pandemic, we were unable to recruit subjects as planned (i.e., 37 per group). Another reason could be that generic instruments (i.e., EQ-5D, and PedsQL) might not be responsive. Instruments such as PedsQL™ stage 5 CKD module could be more sensitive to detect small but clinically significant changes from stage 5 CKD treatment. However, the PedsQL™ stage 5 CKD module has not yet been translated into Thai, while the PedsQL™ 4.0 Generic Core Scales were reported to have acceptable psychometric properties for children of stage 5 CKD [2, 3] and the Thai version of the PedsQL™ 4.0 Generic Core Scales has been validated [22]. In addition, EQ-5D is a generic instrument that covers only 5 dimensions (i.e., mobility, self-care, usual activities, pain/discomfort, and anxiety/depression). It does not cover pediatric-specific domains such as school and social domains that were substantially affected by dialysis treatment.

It should be noted that measuring HRQoL among children was challenging. In our study, we used PedsQL, which is relevant for pediatric patients. The instrument was also developed to fit the specific developmental age of the child. Although PedsQL was available in both the proxy report and the child self-report, previous evidence revealed some discrepancies in inter-rater agreement between child and proxy reporting, especially in psychosocial-related domains [29]. Similar to previous studies [30, 31], we found that the scores reported by the child seem to be higher than those reported by the parent. Although the scores evaluated by parents could not substitute for the perception of HRQoL of the child, they provide parents' perception of their child's well-being that could influence the utilization of health care [32]. Furthermore, dialysis not only imposes a substantial burden on pediatric patients, but also on parents/ caregivers, as these patients often require care and assistance in their daily lives and for dialysis administration. Therefore, caregiver HRQoL should also be evaluated and considered when making a policy on stage 5 CKD treatment.

Our study also reported HRQoL in terms of utility score, which allows the calculation of QALY useful to perform cost-utility analysis. However, measuring utility among children and adolescents was even more challenging [33, 34]. While both EQ-5D-5L and EQ-5D-3L were considered valid and reliable instruments for measuring utility, EQ-5D-5L appeared to have better psychometric properties than EQ-5D-3L [35]. At the time the study was conducted, the Thai Health Technology Assessment Guideline did not clearly recommend the version of the EQ-5D to be used in the economic evaluation study. Furthermore, it was found that the cost-utility results were affected by the change in utility measurements from EQ-5D-3L to EQ-5D-5L [36]. As Thailand has both EQ-5D-5L and EQ-5D-3L value sets, we employed both EQ-5D-5L and EQ-5D-3L to examine the utility scores. This could allow us to compare the cost-utility result when using different utility measurements. Similarly to previous studies, the utility values obtained from EQ-5D-3L were lower than those obtained from EQ-5D-5L [35, 37] and could affect the results of the cost-effectiveness study [38]. However, since recent evidence consistently supported the use of utility scores obtained for EQ-5D-5L over those obtained from EQ-5D-3L [39, 40], the utility score derived from EQ-5D-5L should be applied in the calculation of QALY. 

While a significant improvement in HRQoL can be expected among patients newly started on dialysis due to an improvement in uremic symptoms, it should be noted that our patients were on dialysis for some period of time before participation in the study. Approximately half of the patients were on dialysis for approximately 15 months prior to participation in the study. The median time on dialysis of CAPD was 1.25 years with minimum time of 0.13 years and a maximum time of 6.65 years. On the other hand, the median dialysis time of the APD group was 1.24 years with a minimum time of 0.10 years and a maximum time of 9.0 years. Dialysis time of < 4 months was reported in 27 patients (18 patients in the CAPD group vs. 9 patients in the APD group). However, no significant differences were found between the CAPD and APD groups in terms of dialysis time before participation in the study. Although it may be possible that patients in the APD group who had a very short time on CAPD before participation would experience higher quality of life improvement than those who had a longer time on CAPD before participation in the study, we were unable to explore such impacts due to the limited sample size. In addition, it should be noted that HRQoL scores measured in our study may not be directly generalized to newly started patients with CAPD or APD due to adaptation and cumulative effects of long-term dialysis treatment. Nevertheless, it is important to note that the proportion of patients treated with APD remains low in low- and middle-income countries (LMICs), ranging from 50% in low-income countries to 52% in LMICs [41]. Given that the transition from CAPD to APD is expected to increase [42], our study provides valuable evidence on the HRQoL of patients who switch from CAPD to APD compared to those who continue CAPD.

In addition to the small sample size, which is the main limitation of the study, this present study also has other limitations. First, while EQ-5D-Y, a version of EQ-5D for children and adolescents, is suitable for self-completion by children aged 8–15 years, we did not use the EQ-5D-Y in our study, as there was no value set available for the Thai population. Without the value set, we could not convert the responses from the EQ-5D-Y descriptive system into the utility values. Therefore, the measure of utility in our study was obtained from EQ-5D-5L and EQ-5D-3L using Thai adult value sets. However, the EQ-5D value sets used in this study might not represent the child’s perception, but the perception of the adult. If the value set for EQ-5D-Y becomes available for the Thai population, EQ-5D-Y should be the preferred measure of utility for children and adolescents in the country. Second, while EQ-5D-5L is recommended in children age ≥ 12 years, the questionnaire was administered to children age ≥ 8 years in our study. The total number of children aged ≥ 8 and ≥ 12 years was 51 (28 for the CAPD vs. 23 for the APD group), and 33 (19 for the CAPD vs. 14 APD group), respectively. Due to the cognitive ability of younger children (age 8 to 11 years old), the utilities obtained from EQ-5D-5L assessed by children in our study might be less reliable than those measured by parent-proxy. Furthermore, due to the small sample size, participant withdrawal and a lower completion rate during follow-up, we were unable to identify significant differences in HRQoL, although the magnitude of some differences is larger than MCID. Further studies with a larger sample size and qualitative studies exploring the comprehensive impacts of APD are warranted. In addition, it should also be noted that some participating hospitals contributed only a small number of patients, leading to an imbalance in the number of patients between the APD and CAPD groups across centers, which may have affected the precision of the estimated coefficients.

Nevertheless, all 11 hospitals were tertiary care centers capable of managing pediatric patients with stage 5 CKD. Furthermore, our analysis comparing CAPD and APD across hospital settings, categorized as university hospitals versus non-university tertiary hospitals, found no significant difference.

In addition, it should also be noted that this article focused only on the quality of life outcome, which is the primary outcome. As the impacts of different treatment modalities on secondary outcomes will be reported in subsequent publications, the ability to draw conclusions on the comprehensive impacts of different peritoneal dialysis modalities from this publication might be limited. Meanwhile, such information can be obtained by contacting the authors. Lastly, it should be noted that the HRQoL scores evaluated in our studies could be affected by the COVID-19 pandemic; therefore, the study results may not be generalizable.

In conclusion, no significant benefit of APD over CAPD was found, possibly due to the small sample size and the use of generic instruments. A larger study along with a qualitative study is warranted to examine the comprehensive impact of APD among pediatric patients with stage 5 CKD. Meanwhile, both CAPD and APD should be available to allow patient choice that is consistent with the need, lifestyle, and daily activities of pediatric patients and caregivers, which could improve their HRQoL.

## Supplementary Information

Below is the link to the electronic supplementary material.

Graphical abstract (PPTX 81.5 KB)

Supplementary file2 (DOC 221 KB)

## Data Availability

The datasets generated during and/or analyzed during the current study are available from the corresponding author on reasonable request.
