# Supplementary material for: A comparison of health-related quality of life between continuous ambulatory peritoneal dialysis and automated peritoneal dialysis in children with stage 5 chronic kidney disease in Thailand: a randomized controlled trial
Source: Pediatr Nephrol. 2025 Jan 20;40(6):2029–41. doi: 10.1007/s00467-024-06632-x (PMC12031995; doi:10.1007/s00467-024-06632-x)
Supplement: Supplementary file 1 — Graphical abstract (PPTX 81.5 KB) [file 467_2024_6632_MOESM1_ESM.pptx]

## Slide 1
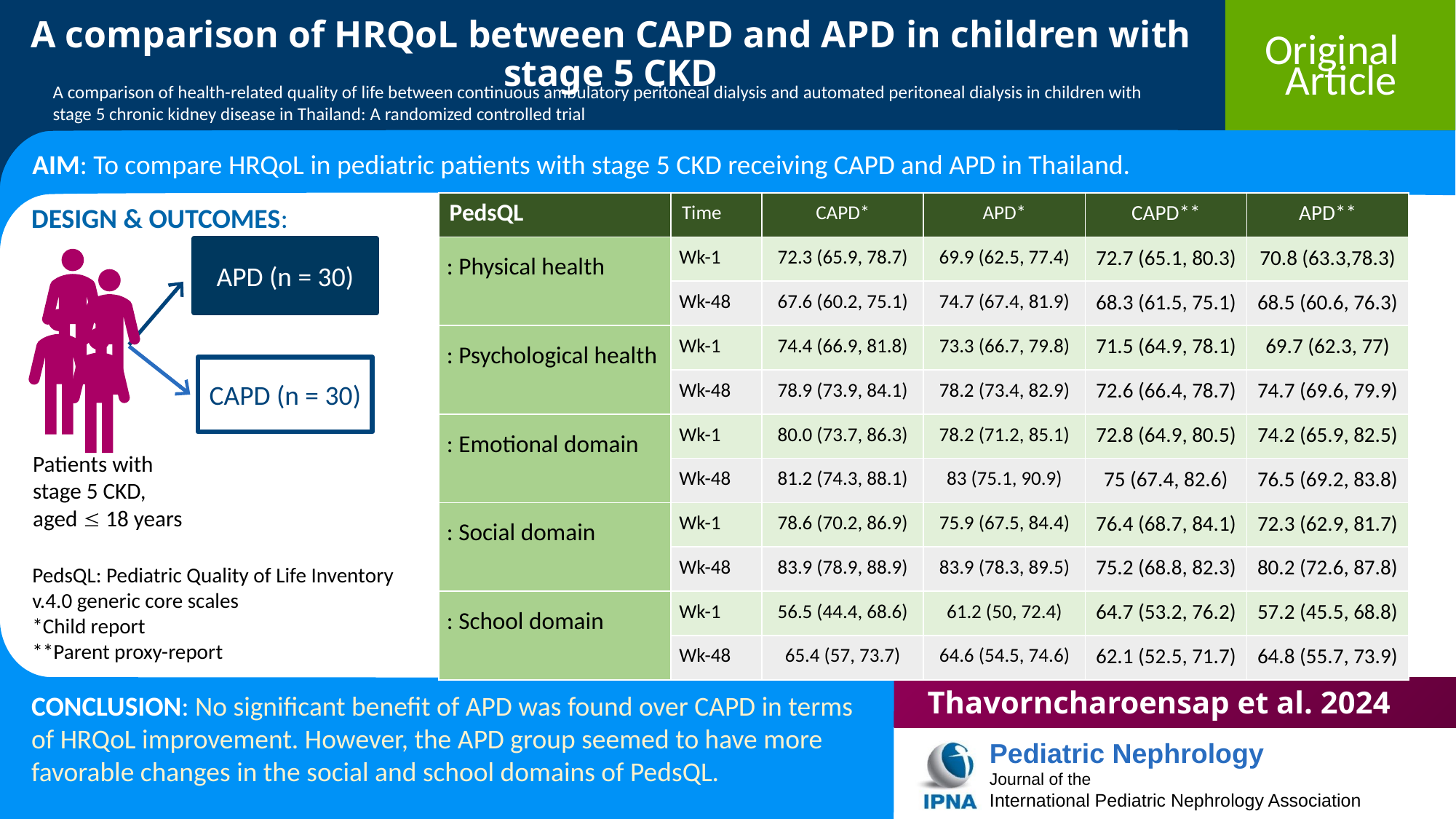

A comparison of HRQoL between CAPD and APD in children with stage 5 CKD
A comparison of health-related quality of life between continuous ambulatory peritoneal dialysis and automated peritoneal dialysis in children with stage 5 chronic kidney disease in Thailand: A randomized controlled trial
AIM: To compare HRQoL in pediatric patients with stage 5 CKD receiving CAPD and APD in Thailand.
| PedsQL | Time | CAPD\* | APD\* | CAPD\*\* | APD\*\* |
| --- | --- | --- | --- | --- | --- |
| : Physical health | Wk-1 | 72.3 (65.9, 78.7) | 69.9 (62.5, 77.4) | 72.7 (65.1, 80.3) | 70.8 (63.3,78.3) |
| | Wk-48 | 67.6 (60.2, 75.1) | 74.7 (67.4, 81.9) | 68.3 (61.5, 75.1) | 68.5 (60.6, 76.3) |
| : Psychological health | Wk-1 | 74.4 (66.9, 81.8) | 73.3 (66.7, 79.8) | 71.5 (64.9, 78.1) | 69.7 (62.3, 77) |
| | Wk-48 | 78.9 (73.9, 84.1) | 78.2 (73.4, 82.9) | 72.6 (66.4, 78.7) | 74.7 (69.6, 79.9) |
| : Emotional domain | Wk-1 | 80.0 (73.7, 86.3) | 78.2 (71.2, 85.1) | 72.8 (64.9, 80.5) | 74.2 (65.9, 82.5) |
| | Wk-48 | 81.2 (74.3, 88.1) | 83 (75.1, 90.9) | 75 (67.4, 82.6) | 76.5 (69.2, 83.8) |
| : Social domain | Wk-1 | 78.6 (70.2, 86.9) | 75.9 (67.5, 84.4) | 76.4 (68.7, 84.1) | 72.3 (62.9, 81.7) |
| | Wk-48 | 83.9 (78.9, 88.9) | 83.9 (78.3, 89.5) | 75.2 (68.8, 82.3) | 80.2 (72.6, 87.8) |
| : School domain | Wk-1 | 56.5 (44.4, 68.6) | 61.2 (50, 72.4) | 64.7 (53.2, 76.2) | 57.2 (45.5, 68.8) |
| | Wk-48 | 65.4 (57, 73.7) | 64.6 (54.5, 74.6) | 62.1 (52.5, 71.7) | 64.8 (55.7, 73.9) |
DESIGN & OUTCOMES:
APD (n = 30)
CAPD (n = 30)
Patients with
stage 5 CKD,
aged ≤ 18 years
PedsQL: Pediatric Quality of Life Inventory v.4.0 generic core scales
*Child report
**Parent proxy-report
Thavorncharoensap et al. 2024
CONCLUSION: No significant benefit of APD was found over CAPD in terms of HRQoL improvement. However, the APD group seemed to have more favorable changes in the social and school domains of PedsQL.
